# Elevated Leukocyte Glucose Index Is Associated with Long-Term Arteriovenous Fistula Failure in Dialysis Patients

**DOI:** 10.3390/jcm13072037

**Published:** 2024-04-01

**Authors:** Adrian Vasile Mureșan, Elena Florea, Emil-Marian Arbănași, Réka Bartus, Eliza-Mihaela Arbănași, Alexandru Petru Ion, Bogdan Andrei Cordoș, Vasile Bogdan Halatiu, Raluca Niculescu, Adina Stoian, Claudiu Constantin Ciucanu, Eliza Russu

**Affiliations:** 1 Department of Vascular Surgery, George Emil Palade University of Medicine, Pharmacy, Science and Technology of Targu Mures, 540139 Targu Mures, Romania; adrian.muresan@umfst.ro (A.V.M.); eliza.russu@umfst.ro (E.R.); 2 Clinic of Vascular Surgery, Mures County Emergency Hospital, 540136 Targu Mures, Romania; 3 Doctoral School of Medicine and Pharmacy, George Emil Palade University of Medicine, Pharmacy, Science and Technology of Targu Mures, 540139 Targu Mures, Romania; 4 Regenerative Medicine Laboratory, Centre for Advanced Medical and Pharmaceutical Research (CCAMF), George Emil Palade University of Medicine, Pharmacy, Science and Technology of Targu Mures, 540139 Targu Mures, Romania; bogdan.cordos@umfst.ro; 5 Centre for Experimental Medical and Imaging Studies, George Emil Palade University of Medicine, Pharmacy, Science and Technology of Targu Mures, 540139 Targu Mures, Romania; 6 Department of Physiology, George Emil Palade University of Medicine, Pharmacy, Science and Technology of Targu Mures, 540139 Targu Mures, Romania; bogdan.halatiu@umfst.ro; 7 Department of Pathophysiology, George Emil Palade University of Medicine, Pharmacy, Science and Technology of Targu Mures, 540139 Targu Mures, Romania; adina.stoian@umfst.ro

**Keywords:** arteriovenous fistula, AVF, dialysis, Leukocyte Glucose Index (LGI), biomarker, vascular access, vascular surgery

## Abstract

(1) **Background**: Arteriovenous fistula (AVF) is the preferred type of vascular access for dialysis in patients with end-stage kidney disease (ESKD). However, the primary patency of AVF at one year is under 70% due to several risk factors and comorbidities. Leukocyte glucose index (LGI), a new biomarker based on blood leukocytes and glucose values, has been found to be associated with poor outcomes in cardiovascular disease. The aim of this study is to analyze the impact of LGI on the long-term primary patency of AVF following dialysis initiation. (2) **Methods**: We conducted a retrospective observational study in which we initially enrolled 158 patients with ESKD admitted to the Vascular Surgery Department of the Emergency County Hospital of Targu Mures, Romania, to surgically create an AVF for dialysis between January 2020 and July 2023. The primary endpoint was AVF failure, defined as the impossibility of performing a chronic dialysis session due to severe restenosis or AVF thrombosis. After follow-up, we categorized patients into two groups based on their AVF status: “functional AVF” for those with a permeable AVF and “AVF failure” for those with vascular access dysfunction. (3) **Results**: Patients with AVF failure had a higher prevalence of atrial fibrillation (*p* = 0.013) and diabetes (*p* = 0.028), as well as a higher LGI value (1.12 vs. 0.79, *p* < 0.001). At ROC analysis, LGI had the strongest association with the outcome, with an AUC of 0.729, and an optimal cut-off value of 0.95 (72.4% sensitivity and 68% specificity). In Kaplan–Meier survival analyses, patients in the highest tertile (T3) of LGI had a significantly higher incidence of AVF failure compared to those in tertile 1 (*p* = 0.019). Moreover, we found that patients with higher baseline LGI values had a significantly higher risk of AVF failure during follow-up (HR: 1.48, *p* = 0.003). The association is independent of age and sex (HR: 1.65, *p* = 0.001), cardiovascular risk factors (HR: 1.63, *p* = 0.012), and pre-operative vascular mapping determinations (HR: 3.49, *p* = 0.037). (4) **Conclusions**: In conclusion, high preoperative values of LGI are positively associated with long-term AVF failure. The prognostic role of the biomarker was independent of age, sex, cardiovascular risk factors, and pre-operative vascular mapping determinations.

## 1. Introduction

Arteriovenous fistula (AVF) is the preferred type of vascular access for dialysis in patients with end-stage kidney disease (ESKD) due to the high long-term functionality and the reduced rate of complications compared to arteriovenous graft (AVG) and central venous catheter (CVC) for dialysis [1]. However, specialists in the field face significant challenges in maintaining long-term AVF patency and managing complications [1,2,3]. According to the European Society of Vascular and Endovascular Surgery (ESVS) guide and studies from the literature, the primary patent of AVF at one year is generally around 70% [1,4,5,6]. There are several factors that affect the maturation process and the permeability of the AVF, including the initiation of dialysis through a central venous catheter for dialysis as indicated in the results of Ravani et al. [7]. Additionally, the systemic inflammatory status, as described in articles published by Kaller et al. [8,9] and the preoperative diameter of the venous component are also contributing factors [8]. Moreover, patients with ESKD have several risk factors that increase the probability of primary AVF failure and death [8,10,11,12]. The most important factors are diabetes mellitus [8,10] and poor glycemic control [11,12].

Drechsler et al. [13] have shown that poor glycemic control is linked to sudden cardiac death in diabetic hemodialysis patients. This finding was based on a study involving 1255 patients [13]. Similarly, Tsujimoto et al. [14] and Tascona et al. [15] found that glycemic control is critical for dialysis patients. Moreover, Reddan et al. [16] and Hsu et al. [17] analyzed and demonstrated the predictive role of white blood cells (leukocytes) in the mortality of dialysis patients. Recently, a new biomarker based on blood leukocytes and glucose values has been proposed in cardiovascular disease [18,19,20,21]. The elevated value of leukocyte-glucose index (LGI) has been found to be associated with the unfavorable evolution of patients with acute myocardial infarction [18,19], the severity of coronary artery disease (CAD) [20,21] and the severity of COVID-19 patients [22].

The aim of this study is to analyze the impact of LGI on the long-term primary patency of AVF following dialysis initiation. Additionally, we will investigate risk factors linked to long-term dysfunction of vascular access.

## 2. Materials and Methods

### 2.1. Study Population

We conducted a retrospective observational study in which we initially enrolled all patients over 18 years with ESKD admitted to the Vascular Surgery Department of the Emergency County Hospital of Targu Mures, Romania, to surgically create an AVF for dialysis between January 2020 and July 2023. We excluded patients with a previous non-functioning or failed AVF, patients who failed to initiate dialysis at the AVF level, as well as patients with hematological diseases, septic conditions, and peripheral arterial disease stage IV Leriche Fontaine. In order to address the current knowledge gap regarding the AVF failure post-initiation of dialysis at the AVF level, we excluded patients who died during the follow-up period. Thus, in the end, 158 patients from the aforementioned period ended up initiating dialysis at the level of the AVF created and fulfilled the inclusion and exclusion criteria. After follow-up, we categorized patients into two groups based on their AVF status: “functional AVF” for those with a permeable AVF and “AVF failure” for those with vascular access dysfunction.

### 2.2. Data Collection

We extracted demographic data such as age and sex, along with various risk factors such as smoking, obesity, and cardiovascular comorbidities, from the hospital’s electronic database. The comorbidities we recorded include hypertension, atrial fibrillation, diabetes, ischemic heart disease, peripheral arterial disease, and prevalent myocardial infarctions and strokes. We also noted whether the AVF occurred in an ambulatory or during continuous hospitalization (lasting more than 24 h). Additionally, we recorded the type of AVF performed as follows: radio-cephalic AVF (RC-AVF), brachio-cephalic AVF (BC-AVF), and brachio-basilic AVF (BB-AVF), as well as whether it was performed at the level of the dominant or non-dominant upper limb.

We collected the arterial diameter, venous diameter, and vein depth data from the hospital’s electronic database, which was determined during the pre-operative mapping. This information was available for 109 patients, of which 90 were in the functional AVF group and 19 were in the AVF failure group.

In terms of laboratory analyses, only pre-operative data was included in the current study. We recorded the following values from the blood count: leukocytes, hemoglobin, hematocrit, neutrophils, lymphocytes, monocytes, and platelets (PLT). Additionally, we recorded the values of glucose, serum albumin, total protein, total calcium, cholesterol, triglycerides, creatinine, blood urea nitrogen (BUN), interleukin-6 (IL-6) available in 91 patients, as well as potassium and sodium values. The LGI was calculated using the following formula [22]:

$$LGI=\frac{Leukocytescounts(\times 10^{3}/\mu L)*glucoselevels(mg/dL)}{1000}$$

### 2.3. Study Outcomes

The primary endpoint was AVF failure, defined as the impossibility of performing a chronic dialysis session due to severe restenosis or AVF thrombosis. In order to record AVF failure rates to avoid imposing unnecessary visits, we requested the status and patency of patients’ AVF from the chronic dialysis centers. The last follow-up date for all patients was 31 December 2023.

### 2.4. Statistical Analysis

SPSS for Mac OS version 28.0.1.0 was used for statistical analysis (SPSS, Inc., Chicago, IL, USA). The age and pre-operative vascular mapping information are presented as mean ± standard deviation (SD). Laboratory data are presented as median (quartile 1-quartile 3). Chi-square tests were used to compare characteristics between the groups for dichotomous variables, while Mann–Whitney and Student’s *t*-test were used to assess differences in continuous variables. We utilized the Spearman correlation to examine the association between LGI and IL-6. The ROC curve analysis was used to determine the appropriate leukocyte, admission glucose level, neutrophils, and LGI cut-off values based on the Youden index (Youden Index = Sensitivity + Specificity − 1, ranging from 0 to 1). We used multivariate Cox proportional hazard analyses to identify independent predictors of AVF failure in ESKD patients. Moreover, HR was expressed per 1 SD increase in the baseline for all laboratory data analyzed. Additionally, we used three different adjustment models to assess the associations between LGI and AVF failure. Thus, Model 1 includes age and sex; Model 2 includes age, sex, and cardiovascular risk factors (diabetes, hypertension, peripheral arterial disease, smoking, obesity); and Model 3 which additionally includes pre-operative vascular mapping (arterial and vein diameter). Kaplan–Meier curves were used to model the crude association between LGI (divided into tertiles) and AVF failure. The Log Rank test was used to compare the curves. All tests were two-tailed, and a *p*-value less than 0.05 was considered statistically significant.

## 3. Results

In the current study, we enrolled 158 patients with an average age of 60.26 ± 14.44. Out of these patients, 62.02% were male. The most common comorbidities found were hypertension in 91.77% of patients, followed by ischemic heart disease in 65.19%, and diabetes in 40.51% of patients (Table 1). Of all the patients, 51.27% were continuously hospitalized, and in 48.73% of the patients, AVF was surgically created in the ambulatory. Following the initiation of dialysis at the AVF level, we monitored the patients for an average of 1.75 ± 1.21 years (Table 1).

Patients with AVF failure had a higher prevalence of atrial fibrillation (20.69% vs. 6.20%, *p* = 0.013), diabetes (58.62% vs. 36.43%, *p* = 0.028), as well as a lower incidence of the male sex, but without significant difference statistically (48.27% vs. 65.11%, *p* = 0.091). Moreover, these patients had higher values of leukocytes (9.18 vs. 7.5, *p* = 0.003), glucose (118 vs. 100.5, *p* = 0.012), neutrophils (5.85 vs. 5.12, *p* = 0.043), and potassium (5.42 vs. 5.10, *p* = 0.008) at baseline (Table 1).

No significant differences were observed between the two groups of patients regarding the type of AVF or the dominant or non-dominant upper limb where the vascular access was performed. We also analyzed the diameter of the artery and vein and the depth of the vein determined by preoperative vascular mapping in 109 patients. According to the results presented in Table 1, we recorded a smaller arterial (2.77 vs. 3.17, *p* = 0.007) and venous (2.71 vs. 3.27, *p* = 0.008) diameter in the patients who experienced AVF failure during follow-up, with no statistically significant difference regarding the depth of the vein.

**Figure 1:**
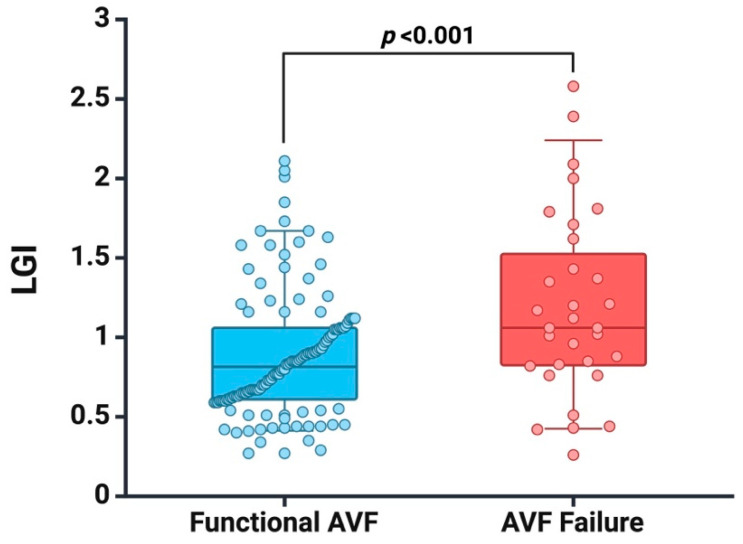
Box plots of baseline LGI in patients with functional AVF and AVF failure at follow-up. Created with BioRender.com (accessed on 10 January 2024).

To assess systemic inflammatory status, we measured IL-6 levels (*n* = 91 patients) and found higher IL-6 values (9.15 vs. 5.48, *p* < 0.001) in patients who experienced AVF failure. Furthermore, we recorded a higher LGI value in patients with AVF failure (1.12 vs. 0.79, *p* < 0.001), as seen in Figure 1.

**Figure 2:**
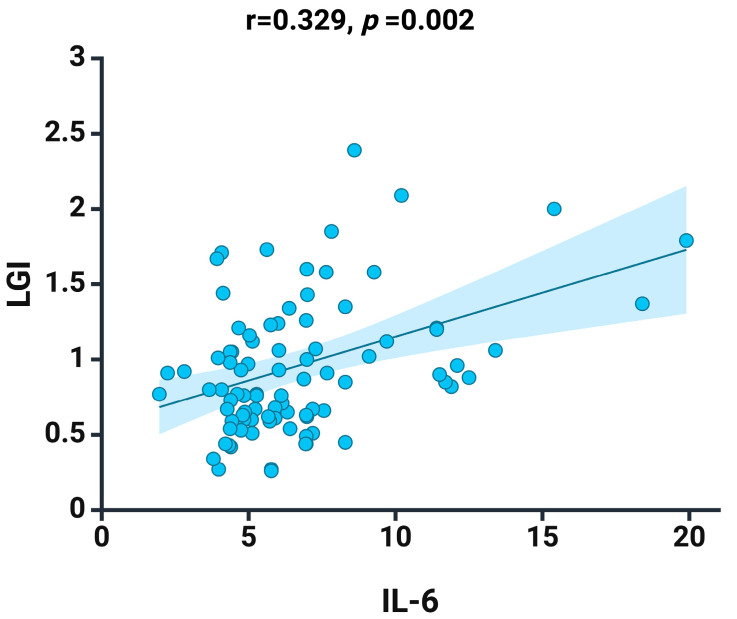
Spearman correlation between LGI and the IL-6 at baseline. Graphs depict a linear fitted line describing the dataset. Shaded area represent 95% confidence interval. Created with BioRender.com (accessed on 3 March 2024).

We recorded a positive correlation between IL-6 and LGI (r = 0.329, *p* = 0.002) (Figure 2), an aspect that argues the effectiveness of this new biomarker proposed and analyzed by us in the current study.

In the ROC analysis, we found that leukocytes (*p* = 0.001), glucose level (*p* = 0.010), neutrophils (*p* = 0.013), and LGI (*p* < 0.001) are linked with AVF failure (Table 2). According to our findings, LGI had the strongest association with an AUC of 0.729, and an optimal cut-off value of 0.95 was recorded, with 72.4% sensitivity and 68% specificity. Moreover, leukocyte count has an optimal cut-off value of 8.14 with a sensitivity of 75.9% and specificity of 63.6%. Glucose has an optimal cut-off value of 103.25 with 72.4% sensitivity and 54.5% specificity, while neutrophils have an optimal cut-off value of 4.94 with 72.4% sensitivity and 48.1% specificity.

**Figure 3:**
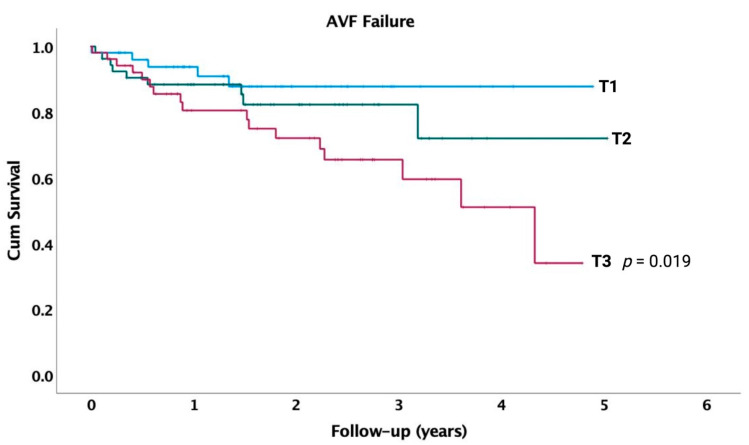
Survival curves for incident AVF failure in entire cohort, by tertiles of LGI at baseline. *p*-value indicates difference between respective tertiles and lowest tertile (T1), calculated using unadjusted log-rank test.

In Kaplan–Meier survival analyses with log-rank test, patients in the highest tertile (T3) for LGI had a significantly higher incidence of AVF failure compared to those in tertile 1 (*p* = 0.019) (Figure 3). We conducted multivariate Cox proportional hazard analyses to investigate the association between risk factors, laboratory data, and incidence of AVF failure (Table 3). Thus, patients with atrial fibrillation have a three-times higher risk of AVF failure (HR: 3.10, *p* = 0.014) compared to those without atrial fibrillation. Additionally, higher baseline values of glucose are linked with an increased risk of AVF failure (HR: 1.35, *p* = 0.018).

Moreover, we found that patients with higher baseline LGI values had a significantly higher risk of AVF failure during follow-up (HR: 1.48, *p* = 0.003). The association is independent of age and sex (HR: 1.65, *p* = 0.001), cardiovascular risk factors (HR: 1.63, *p* = 0.012), and pre-operative vascular mapping determinations (HR: 3.49, *p* = 0.037; *n* = 109 patients) (Table 4).

## 4. Discussion

The current study results demonstrate, for the first time to our knowledge, the positive association between higher baseline LGI values and long-term AVF failure. The prognostic role of LGI is independent of age, sex, cardiovascular risk factors, or pre-operative vascular mapping determinations. Additionally, patients with atrial fibrillation have a three-times higher risk of AVF failure. Furthermore, patients with vascular access dysfunction are more likely to have diabetes, although this relationship loses its statistical significance in cox-regression analyses. We found a positive correlation between LGI and IL-6 in 91 patients of the entire cohort where we had the IL-6 value available. This finding supports the effectiveness of the new biomarker proposed and analyzed in our current study.

Several studies have shown that female patients are at a higher risk of experiencing early and long-term failure of AVF compared to male patients [23,24,25]. For instance, a study by Hernández et al. [23] on 119 patients with 148 native AVFs found that female patients were four-times more likely to experience early failure (adjusted odds ratio: 4.04, *p* = 0.008). Another study by Miller et al. [24] observed that female patients had lower functionality rates for both forearm AVF (18% vs. 43%, *p* = 0.02) and upper arm AVF (39% vs. 60%, *p* = 0.04). However, two other studies by Okamuro et al. [26] and Voorzaat et al. [27] did not find any significant association between demographic parameters and AVF failure in a larger cohort of patients. Similarly, in our study, although we observed a lower prevalence of male patients among those who experienced AVF failure, there was no difference between male and female patients. These findings corroborate other recently published articles by Kaller et al. [8,9] and Mureșan et al. [10], where the authors observed no gender-related differences in AVF maturation rate [8,9] and mortality among ESKD patients [10].

Previous clinical studies have demonstrated that the presence of diabetes increases the risk of AVF failure [11,28,29,30,31]. Park et al. [28] identified in the Cox proportional hazard model analysis that diabetes is an independent risk factor of primary patency (HR: 2.008, *p* = 0.043) in a cohort of 383 autogenous AVF. Similarly, Cheng et al. [29] observed that patients with diabetes accounted for a higher percentage of cases where the primary AVF surgery failed (46.8% vs. 31.7%, *p* = 0.023). These findings are also supported by a meta-analysis published by Yan et al. [30]. In our cohort, although there was a higher prevalence of diabetes in patients with AVF failure during follow-up (58.62% vs. 36.43%, *p* = 0.028), it was not a predictive factor of vascular access dysfunction. However, Roan et al. [32] found that rats with diabetes have lower AVF blood flow than rats without diabetes, as well as upregulated protein expression of inducible nitric oxide synthase. These findings indicate that blood flow in the AVF of diabetic individuals is reduced because of the activation of proinflammatory genes.

The potential of LGI as a biomarker in predicting cardiovascular pathologies has been studied extensively in recent years [18,19,20,21,33,34,35,36,37]. León-Aliz et al. [33] first proposed LGI as a biomarker to predict in-hospital mortality in patients with ST-segment elevation myocardial infarction (MI) in 2014 (OR: 3.0, *p* = 0.005). Sadeghi et al. [19], Qi et al. [34], and Reyes-Villarreal et al. [35] validated the role of LGI in predicting in-hospital mortality [19,34], major adverse cardiovascular and cerebrovascular events [34], and non-cardiovascular complications [35] in three cohorts of patients with MI. Kilic et al. (21) and Demir et al. [36] also found that LGI values can predict the severity of CAD (HR: 1.003, *p* = 0.002) and multivessel CAD (OR: 1.599, *p* = 0.018), respectively. This new biomarker, calculated based on the leukocyte count and glucose level, shows promising results in predicting cardiovascular disease. Moreover, the current study found that high LGI values can predict long-term AVF failure independent of age, sex, cardiovascular risk factors, and pre-operative vascular mapping determinations. Since LGI is easy to determine and has low cost, its future introduction in the management of patients would allow for better stratification of risk groups.

Several studies have shown that inflammatory biomarkers based on blood cell counts of neutrophils, monocytes, platelets, and lymphocytes can predict AVF dysfunction [8,9,10] and long-term mortality in dialysis patients [10,16,17]. When the venous wall is exposed to arterial pressure, it undergoes a histological remodeling process that results in the proliferation, differentiation, and migration of smooth muscle cells (SMC), generating long-term intimal hyperplasia (IH) [38,39]. This process is mediated by various factors, including insulin-like growth factor-1 (IGF-1) [40], which also plays a role in the mortality of dialysis patients [41]. Moreover, IGF-1 regulates glucose levels and lipid metabolism in diabetic patients [42]. These findings suggest that IH and AVF dysfunction might be related to poor glycemic control and the pro-inflammatory effects of white blood cells. Additionally, since the biomarker may be linked to IH, we recommend that patients with a baseline LGI value above the optimal cut-off identified in our study should undergo regular ultrasound monitoring and their glycemic control should be checked.

In the current study, it was found that there is a positive relationship between LGI and IL-6 (r = 0.329, *p* = 0.002). In a study conducted by Baek et al. [43], it was demonstrated that patients who had IL-6 values in tertile 3 were three times more likely to experience AVF dysfunction at the 12-month mark (HR: 3.06, *p* = 0.015). Moreover, several articles have shown that high levels of IL-6 are linked with mortality in dialysis patients [44,45,46,47]. According to Panichi et al. [48], IL-6 is a stronger predictor of total and cardiovascular mortality than C-reactive protein. Additionally, Marrone et al. [49] have found that the activation of the IL-6 receptor plays a role in AVF failure. Despite the positive results found in the literature [1,2,3,4,5,6], the high cost and the difficulty of determining IL-6 values for all hospitalized patients to create vascular access have prevented the inclusion of this biomarker in current medical practice.

Recently, Russu et al. [50] published a review presenting therapeutic strategies based on non-ionizing radiation that have shown interesting results in improving AVF functionality. Photodynamic therapy has been demonstrated in animal models of AVF to inhibit the development of IH [51,52,53,54]. Far infrared therapy has had the best results in human clinical trials, improving AVF flow, AVF maturation, and primary and secondary patency [55,56,57,58,59,60]. Additionally, recent research has shown that photo-crosslinking of adventitial collagen fibers reduces venous wall compliance, inhibits AVF aneurysmal development [61], and decreases the risk of abdominal aortic aneurysm rupture [62].

It is important to note that our study has several limitations. Firstly, our research indicates for the first time the connection between high preoperative LGI values and long-term AVF failure in a monocentric small cohort of 158 patients with ESKD. Further studies are required to validate our findings, along with prospective multicenter studies that enable the determination of an optimal threshold value. Secondly, we were unable to analyze the relationship between the biomarker and IH at the AVF level due to the lack of ultrasound determination of IH severity. Thirdly, another important limitation of our study is that we included only data from pre-operative vascular mapping in 109 patients. Since our study was retrospective, we did not have the pre-operative arterial flow recorded in the hospital’s electronic database for all patients in the cohort. Moreover, due to the retrospective design, there is insufficient data on cannulation technique and timing of the first cannulation, as well as infection control practices to be included in the current analysis. Lastly, our study only examined pre-operative LGI values and did not track their dynamic evolution at specific intervals. Thus, we recommend following the biomarker’s longitudinal evolution and its impact on AVF permeability in future studies.

## 5. Conclusions

In conclusion, we presented the positive association between high preoperative values of LGI and long-term AVF failure for the first time. The prognostic role of the biomarker was independent of age, sex, cardiovascular risk factors, and pre-operative vascular mapping determinations. We also identified a higher prevalence of atrial fibrillation and ambulatory AVF surgery in patients with vascular access dysfunction. LGI can be used as a potential biomarker to identify patients from risk groups that require more careful monitoring of AVF in order to improve the management and care of patients with ESKD. This biomarker has shown promising results in other cardiovascular pathologies, such as myocardial infarction and the severity of coronary artery disease. Therefore, new clinical and experimental studies should be conducted to validate its effectiveness.

## Figures and Tables

**Table 1:** Baseline differences in clinical characteristics between patients with and without AVF failure during follow-up.

Variables	All Patients *n* = 158	Functional AVF *n* = 129	AVF Failure *n* = 29	*p* Value
Age mean ± SD	60.26 ± 14.44	59.88 ± 14.78	61.93 ± 12.93	0.457
Male gender no. (%)	98 (62.02%)	84 (65.11%)	14 (48.27%)	0.091
Comorbidities and Risk factors, no. (%)				
Hypertension	145 (91.77%)	119 (92.25%)	26 (89.66%)	0.646
Atrial Fibrillation	14 (8.86%)	8 (6.20%)	6 (20.69%)	0.013
Diabetes	64 (40.51%)	47 (36.43%)	17 (58.62%)	0.028
Ischemic Heart Disease	103 (65.19%)	81 (62.79%)	22 (75.86%)	0.182
Peripheral Arterial Disease	22 (13.92%)	16 (12.4%)	6 (20.69%)	0.244
Prevalent Myocardial Infarction	9 (5.70%)	6 (4.65%)	3 (10.34%)	0.232
Prevalent Stroke	9 (5.70%)	7 (5.43%)	2 (6.90%)	0.758
Smoking	15 (9.49%)	13 (10.08%)	2 (6.90%)	0.597
Obesity	32 (20.25%)	26 (20.16%)	6 (20.69%)	0.948
Laboratory data, median (Q1–Q3)				
Leukocytes ×10^3^/μL	7.85 (6.28–9.43)	7.5 (6.13–9.02)	9.18 (7.32–10.2)	0.003
Potassium mmol/l	5.19 (4.65–5.66)	5.10 (4.55–5.56)	5.42 (4.97–6.01)	0.008
Sodium mmol/l	139.6 (137–141)	140 (137–142)	139 (137.5–140)	0.257
Glucose (mg/dL)	103 (89–133)	100.5 (89–126.97)	118 (92.15–163.4)	0.012
BUN (mg/dL)	134.82 (98.77–177.95)	141.9 (99.65–182.95)	122.4 (89.9–168)	0.319
Creatinine (mg/dL)	6.81 (5.47–9.38)	6.84 (5.23–9.48)	6.52 (6.06–7.66)	0.754
Hemoglobin g/dL	10.1 (8.55–11.25)	9.98 (8.47–11)	10.6 (9.83–11.6)	0.119
Hematocrit %	30.6 (26.66–34.77)	30.4 (26.3–34.51)	33 (29.6–36.1)	0.181
Neutrophils ×10^3^/μL	5.26 (4.15–6.55)	5.12 (4.07–6.42)	5.85 (4.82–6.98)	0.043
Lymphocytes ×10^3^/μL	1.41 (1.05–1.93)	1.38 (1.03–1.89)	1.52 (1.16–2.122)	0.075
Monocyte ×10^3^/μL	0.57 (0.44–0.75)	0.57 (0.43–0.73)	0.61 (0.48–0.79)	0.087
PLT ×10^3^/μL	222.8 (181.5–283.37)	223.0 (181–281.35)	221.0 (191.1–306)	0.149
Serum Albumin g/dL	3.6 (3.35–4.01)	3.6 (3.35–4.02)	3.36 (3.3–3.48)	0.177
Total Protein g/dL	6.44 (5.99–7.07)	6.42 (5.94–6.92)	6.95 (6.16–7.81)	0.111
Total Calcium mmol/L	2.10 (1.93–2.22)	2.11 (1.93–2.27)	1.64 (1.59–1.91)	0.178
Cholesterol mg/dL	160.55 (131.62–184.62)	161.5 (133.05–192)	154.0 (129.3–181.1)	0.860
Triglyceride mg/dL	142.25 (121.65–199.2)	150.5 (120.7–201.8)	134.0 (124.3–192.4)	0.441
Interleukin-6 pg/mL *	5.90 (4.7–7.6)	5.48 (4.41–6.95)	9.15 (7.41–11.52)	<0.001
Pre-Operative Vascular Mapping ^#^, mean ± SD				
Arterial diameter (mm)	3.11 ± 1.05	3.17 ± 0.98	2.77 ± 1.30	0.007
Vein diameter (mm)	3.17 ± 0.82	3.27 ± 0.83	2.71 ± 0.65	0.008
Vein depth (mm)	2.69 ± 0.99	2.72 ± 0.94	2.63 ± 1.21	0.317
AVF type and placement, no. (%)				
RC-AVF	74 (46.83%)	62 (48.06%)	12 (41.37%)	0.513
BC-AVF	67 (42.40%)	54 (41.86%)	13 (44.82%)	0.770
BB-AVF	17 (10.75%)	13 (10.07%)	4 (13.79%)	0.561
Dominant Limb	31 (19.62%)	26 (20.15%)	5 (17.24%)	0.721
Non-Dominant Limb	127 (80.38%)	103 (79.85%)	24 (82.76%)	
Ambulatory AVF, no. (%)	77 (48.73%)	56 (43.41%)	21 (72.41%)	0.006
Hospitalization AVF, no. (%)	81 (51.27%)	73 (56.59%)	8 (27.59%)	
Follow-up period (years) mean ± SD/max	1.75 ± 1.21/5.03	1.89 ± 1.17/5.03	1.18 ± 1.77/5.02	0.001

* Value of Interleukin-6 is available only for a group of 91 patients from entire cohort. Among patients who had functional AVF at follow-up, 71 reported IL-6 value, whereas 20 patients in group with AVF failure reported it. ^#^ Pre-operative vascular diameters were available only for 109 patients from entire cohort. Therefore, we have data for 90 patients in group with functional AVF at follow-up, and only for 19 patients in case of those with AVF failure.

**Table 2:** Areas under curve (AUC), cut-off value, sensitivity, specificity for white blood cell count, glucose levels, neutrophils, and LGI in terms of AVF failure.

Variables	Cut-Off	AUC	Std. Error	95% CI	Sensitivity	Specificity	*p* Value
	AVF Failure						
Leukocytes ×10^3^/μL	8.14	0.702	0.052	0.599–0.802	75.9%	63.6%	0.001
Glucose	103.25	0.653	0.056	0.543–0.762	72.4%	54.5%	0.010
Neutrophils ×10^3^/μL	4.94	0.648	0.056	0.538–0.759	72.4%	48.1%	0.013
LGI	0.95	0.729	0.051	0.630–0.829	72.4%	68%	<0.001

**Table 3:** Correlations between comorbidities, baseline laboratory data, and follow-up AVF failure.

Variables	AVF Failure		
	HR *	95% CI	*p* Value
Male	0.51	0.24–1.06	0.073
Atrial Fibrillation	3.10	1.25–7.68	0.014
Diabetes	1.91	0.90–4.04	0.092
Leukocytes ×10^3^/μL	1.26	0.91–1.74	0.176
Glucose (mg/dL)	1.35	1.05–1.73	0.018
Neutrophils ×10^3^/μL	1.17	0.84–1.64	0.337
Lymphocytes ×10^3^/μL	1.33	0.93–1.91	0.116
Monocyte ×10^3^/μL	1.07	0.78–1.48	0.659

* HR expressed per 1 SD increase in baseline for laboratory data (leukocytes, glucose, neutrophils, lymphocytes, and monocytes).

**Table 4:** Cox-regression analysis: association of LGI at baseline and AVF failure during follow-up.

Biomarker	Model	AVF Failure		
		HR	95% CI	*p* Value
LGI	Model 1	1.48	1.14–1.92	0.003
	Model 2	1.65	1.22–2.22	0.001
	Model 3	1.63	1.12–2.38	0.012
	Model 4 *	3.49	1.08–11.34	0.037

* HR expressed per 1 SD increase in baseline LGI. Model 1: unadjusted. Model 2: age and sex. Model 3: age, sex, CV risk factors (diabetes, hypertension, peripheral arterial disease, smoking, obesity). Model 4: age, sex, CV risk factors (diabetes, hypertension, peripheral arterial disease, smoking, obesity), pre-operative vein and artery diameter. Model was calculated for patients with available pre-operative artery and vein diameter data (*n* = 109).

## Data Availability

The raw data supporting the conclusions of this article will be made available by the authors on request.
